# Decreased Default Mode Network connectivity correlates with age-associated structural and cognitive changes

**DOI:** 10.3389/fnagi.2014.00256

**Published:** 2014-09-25

**Authors:** Didac Vidal-Piñeiro, Cinta Valls-Pedret, Sara Fernández-Cabello, Eider M. Arenaza-Urquijo, Roser Sala-Llonch, Elisabeth Solana, Núria Bargalló, Carme Junqué, Emilio Ros, David Bartrés-Faz

**Affiliations:** ^1^Departament de Psiquiatria i Psicobiologica Clinica, Facultat de Medicina, Universitat de BarcelonaBarcelona, Spain; ^2^Unitat de Lípids, Servei Endicronologia i Nutrició, Hospital ClínicBarcelona, Spain; ^3^Laboratoire de neuropsychologie, INSERM U1077Caen, France; ^4^Institut d’Investigacions Biomédiques August Pi i Sunyer (IDIBAPS)Barcelona, Spain; ^5^Servei de Radiologia, Hospital Clínic de BarcelonaBarcelona, Spain

**Keywords:** Default Mode Network, resting-state fmri, memory, aging, connectivity, gray matter, white matter, arterial spin labeling

## Abstract

Ageing entails cognitive and motor decline as well as brain changes such as loss of gray (GM) and white matter (WM) integrity, neurovascular and functional connectivity alterations. Regarding connectivity, reduced resting-state fMRI connectivity between anterior and posterior nodes of the Default Mode Network (DMN) relates to cognitive function and has been postulated to be a hallmark of ageing. However, the relationship between age-related connectivity changes and other neuroimaging-based measures in ageing is fragmentarily investigated. In a sample of 116 healthy elders we aimed to study the relationship between antero-posterior DMN connectivity and measures of WM integrity, GM integrity and cerebral blood flow (CBF), assessed with an arterial spin labeling sequence. First, we replicated previous findings demonstrating DMN connectivity decreases in ageing and an association between antero-posterior DMN connectivity and memory scores. The results showed that the functional connectivity between posterior midline structures and the medial prefrontal cortex was related to measures of WM and GM integrity but not to CBF. Gray and WM correlates of anterio-posterior DMN connectivity included, but were not limited to, DMN areas and cingulum bundle. These results resembled patterns of age-related vulnerability which was studied by comparing the correlates of antero-posterior DMN with age-effect maps. These age-effect maps were obtained after performing an independent analysis with a second sample including both young and old subjects. We argue that antero-posterior connectivity might be a sensitive measure of brain ageing over the brain. By using a comprehensive approach, the results provide valuable knowledge that may shed further light on DMN connectivity dysfunctions in ageing.

## Introduction

As demographic changes in developed countries push up the proportion of elderly adults in the population (Cohen, 2003), age-related cognitive decline is emerging as a major concern. Ageing entails cognitive and motor decline and is the main risk factor for neurodegenerative disorders, especially Alzheimer’s Disease (AD; Hebert et al., 2003). Cognitive domains affected by age include speed of processing, working memory capacity, inhibitory function and long-term episodic memory (Park and Reuter-Lorenz, 2009; Salthouse, 2010). Neuroimaging studies have contributed to the understanding of the ageing brain and have classically associated the elderly with gray mater (GM) shrinkage (Good et al., 2001; Salat et al., 2004; Fjell et al., 2009a,b), ventricular expansion (Walhovd et al., 2011), decreased WM (WM) integrity (Davis et al., 2009; Bennett et al., 2010; Westlye et al., 2010; Sala et al., 2012) and neurotransmitter depletion, particularly of the dopaminergic system (Reeves et al., 2002).

GM atrophy in both cortical and subcortical structures, evident as early as middle age (Salat et al., 2004), has been demonstrated in both cross-sectional (Good et al., 2001; Fjell et al., 2009b) and longitudinal evidences (Raz et al., 2004; Fjell et al., 2009a). Grey matter atrophy analyses, carried out either with Cortical Thickness (CTh) or with Voxel-based morphometry (VBM) approaches, consistently indicate that prefrontal cortices and most subcortical structures are regions of high age-related vulnerability (Good et al., 2001; Fjell et al., 2009b). Lateral and medial temporal lobes and posterior midline structures are also significantly affected by age-related atrophy (Fjell et al., 2014a). Most structures show a linear decline, though specific structures such as the hippocampus may exhibit an increased rate of atrophy with ageing (Fjell et al., 2013).

Both macrostructural and microstructural WM alterations that includes damage in the myelin sheath and reduction in the total number of nerve fibers have been widely reported in aged humans (Tang et al., 1997; Bartzokis et al., 2004; Davis et al., 2009; Westlye et al., 2010; Sala et al., 2012). These changes are believed to impair the efficiency of communication between neural regions and to contribute to the functional decline in elders (Bartzokis et al., 2004). Diffusion Tensor Imaging (DTI) techniques are sensitive to the degree and direction of water molecule permeability and are able to characterize microstructural properties of WM *in vivo*. Increased Fractional Anisotropy (FA) and reduced Mean Diffusivity (MD) are the most frequently used DTI measures associated with WM integrity, as they are able to provide summarized information on the state of WM. Diffusion Tensor Imaging measures are altered over the lifespan, presenting an inverted U-shape curve and accelerated decreases during senescence (Westlye et al., 2010; Sala et al., 2012). Regarding the pattern of age-related differences in DTI measures across brain regions, a variety of models haven been proposed that include frontal, anterior to posterior gradient and retrogenesis, last-in first-out, models (Bennett and Madden, 2013). It has also been suggested that thin myelinated fibers, near the brain surface or on the periphery of fasciculi may be more vulnerable to age-related degradation than deeper structures (Tang et al., 1997; Bartzokis et al., 2004). In addition, a measure that might provide information on both GM and WM atrophy near the cortical surface is the GM/WM contrast (GWC). Several studies have found striking reductions in GWC during ageing (Magnaldi et al., 1993; Salat et al., 2009; Westlye et al., 2009) indicating that GM and WM tissue becomes less differentiated as the brain ages. Gray/white matter contrast alteration is particularly notable in medial and lateral prefrontal areas, inferior and posterior midline parietal areas and lateral temporal areas. A microstructural basis for the blurring of the GM/WM boundary might reflect several processes in ageing, among them alterations in the structure and density of myelin sheaths (Cho et al., 1997), changes in iron concentration (Ogg and Steen, 1998) and/or increased content of water in WM (Magnaldi et al., 1993).

It is widely accepted that ageing and vascular processes interact to disrupt cerebral hemodynamics (de la Torre, 2012). Additionally, neurovascular unit (involving endothelial cells, myocites, neurons and its processes and astrocytes amongst others) is involved in cerebral hemodynamics while preservation of this hemodynamics processes is vital for neural activity and cognitive function (Popa-Wagner et al., 2013). Cerebral blood flow (CBF), classically assessed with radio-ligand based neuroimaging techniques and closely correlated with brain metabolism is a cerebral hemodynamic process known to be reduced in ageing (Martin et al., 1991), vital for optimal neural function and it has been identified as a contributor to cognitive impairment in older adults (Brown and Thore, 2011; Gorelick et al., 2011). Arterial Spin labeling (ASL) is a noninvasive technique able to assess tissue perfusion by magnetic labeling of arterial blood water and have aroused considerable interest in recent years. Arterial Spin labeling measures are affected by ageing (Asllani et al., 2009), even when corrected for partial volume effects (PVE), however systematic studies are needed to fully characterize CBF values over the lifespan and to determine its topological pattern.

More recently, though, brain connectivity at rest has consistently been found to be altered in ageing using the fMRI technique (rs-fMRI; Hafkemeijer et al., 2012; Ferreira and Busatto, 2013). rs-fMRI is able to detect interregional correlations in low-frequency spontaneous BOLD fluctuations (Biswal et al., 1995) which have shown to be a key feature in healthy brain functioning and are altered in multiple neuropsychiatric pathologies (Broyd et al., 2009). A significant finding in rs-fMRI ageing literature is the observation of decreased long-range functional connectivity in elders (Meunier et al., 2009; Tomasi and Volkow, 2012) usually complemented by increased local clustering (Tomasi and Volkow, 2012; Sala-Llonch et al., 2014). Age-related decreases in rs-fMRI mainly affect the Default Mode Network (DMN; Andrews-Hanna et al., 2007; Bluhm et al., 2008; Damoiseaux et al., 2008; Razlighi et al., 2014), a resting state network (RSN) that comprises several structures including posterior midline structures (precuneus/posterior cingulate cortex [PCU/PCC]), medial prefrontal cortex (mPFC), inferior parietal lobule (IPL), and middle and medial (entorhinal/hippocampus) temporal cortex (Laird et al., 2009). Default Mode Network recruitment has been directly associated with episodic memory retrieval, prospective memory, self-referential processes, social cognition or mind wandering and it is usually deactivated during external-oriented tasks (Anticevic et al., 2012), a process strongly affected in ageing (Miller et al., 2008). The interest in the DMN, though, also arises in pathological ageing as connectivity within this network is strongly decreased in Alzheimer’s Disease (AD; Jones et al., 2011) and most of its nodes are key cores of the pathology (Buckner et al., 2009).

The DMN appears to be more susceptible to the effects of ageing (Damoiseaux et al., 2008) than other RSN and this susceptibility may be evident even in middle-age healthy subjects (Bluhm et al., 2008; Biswal et al., 2010; Evers et al., 2012). Moreover, age-related reductions in DMN nodes seems to involve coupling between mPFC and posterior midline structures in particular, which are the key nodes of the DMN (Andrews-Hanna et al., 2007; Bluhm et al., 2008; Biswal et al., 2010; Campbell et al., 2013; Mevel et al., 2013). Default Mode Network rs-fMRI connectivity predicts cognitive and behavioral measures, especially memory function, both in different populations including healthy young subjects (Sala-Llonch et al., 2012) and healthy elders (Andrews-Hanna et al., 2007; Damoiseaux et al., 2008; Wang et al., 2010; He et al., 2012; Mevel et al., 2013; Razlighi et al., 2014). Alterations in the DMN, a core brain network, have been attributed to inefficient reallocation of resources, though the exact causes of decreased DMN are still unknown.

While preserved cerebral connectivity patterns seem to sustain healthy brain functioning, other physiological brain measures appear to modulate connectivity and may provide useful insights into age-related connectivity changes. Perfusion and connectivity have been linked in young adults (Liang et al., 2013), while, during ageing, Aβ deposition (Hedden et al., 2009) and the dopaminergic system (Achard and Bullmore, 2007) have been related to brain functional connectivity. In contrast, local GM atrophy does not seem to fully explain rs-fMRI connectivity changes occurring in ageing (Damoiseaux et al., 2008). However GM functional and structural covariance patterns are highly related (Segall et al., 2012) and relationship between connectivity patterns and GM topology (Seeley et al., 2009) and integrity (Pujol et al., 2013; Baggio et al., 2014) have been shown in several disorders. In contrast it is increasingly accepted that anatomical connectivity supports functional connectivity, which, at rest, is especially tangible in key brain nodes such as those belonging to the DMN (Skudlarski et al., 2008; Honey et al., 2009; Horn et al., 2013). Indeed, relationships between WM integrity indices and functional connectivity measures have been reported in several populations (van den Heuvel et al., 2008; Khalsa et al., 2013) including healthy elderly adults (Andrews-Hanna et al., 2007; Teipel et al., 2010) emerging in the late childhood (Supekar et al., 2010; Gordon et al., 2011). The coupling strength between DMN nodes seems to be supported by the cingulum bundle integrity in ageing which connects posterior to anterior and temporal DMN areas (Greicius et al., 2009).

To gain further insight into the processes underlying age-related brain, the relationships between multiple neuroimaging measures need to be assessed. These experimental settings do not allow any inference of causality between the changes evaluated using different modalities, but can broaden our understanding of the physiological mechanisms underlying age-related changes (Chételat et al., 2013b). Studying the relationship of DMN with other neuroimaging modalities may help to unify disparate findings of the neuroimaging literature and provide a more comprehensive description of altered connectivity in ageing. So far, despite almost unequivocal evidence that DMN connectivity is highly vulnerable to the effects of aging, our understanding of the causes and consequences of decreased antero-posterior DMN connectivity remains limited. Studies investigating structural correlates of DMN integrity in ageing have been limited to specific bundles and to local GM integrity, and little is known about how age-related connectivity changes relate to brain patterns of reduced grey and WM integrity and CBF in ageing.

The main objective of this study was to assess the relationship between mPFC-PCU DMN connectivity and CBF, WM and GM correlates in elders. The specific hypotheses were: (1) mPFC-PCU connectivity will be reduced in ageing; (2) mPFC-PCU connectivity will correlate with cognitive performance in elders, essentially with memory function; (3) in ageing, mPFC-PCU connectivity will correlate with grey and WM integrity as well as with perfusion measures; these relationships will be found in, but not limited to, DMN areas and cingulum bundle; (4) regions correlating with mPFC-PCU coupling strength will be located in areas of high age-related vulnerability. To test these hypotheses we used two independent samples. One large sample of healthy elders with small age-related variability was used to extract structural, perfusion and cognitive correlates of mPFC-PCU coupling strength, while a second sample including young and old subjects was used for purposes of group (age-related) comparisons.

## Materials and methods

### Participants

One hundred and sixteen healthy old subjects (age = 68.25 (3.053); range: 63–78, 37 males) were included in this study. Mean years of education (YoE) was 11.09 (4.149). Subjects were recruited in retirement homes and centers for the elderly registered with the Institut Català del Envelliment (ICN) in the area of Barcelona. All participants had normal cognitive profile with Mini-mental State Examination test (MMSE) scores ≥25 and performances not below 1.5SD according to normative scores adjusted for age, gender and education in a neuropsychological evaluation that covered the major cognitive domains. Informed consent was obtained from all participants. The study was approved by the Hospital Clinic de Barcelona ethical committee which follows the guidelines of the Declaration of Helsinki. All subjects underwent MRI acquisition (DTI data were available for 100 subjects). This aged sample was used to assess the relationship between rs-fMRI connectivity on the one hand, and cognition, structural and perfusion indices on the other and will be referred as *sample 1*.

A second, independent, sample (referred as *sample 2*) consisting of 25 (8 males) young and 25 (8 males) old healthy subjects was used for direct age-groups comparison purposes. Magnetic resonance imaging data from this sample was obtained using exactly the same scanner and sequences as in the primary sample. Mean age was 23.08 (2.02; range 19–28) and 68.92 (3.67; range 64–76) for the young and old group respectively. Mean YoE was 19.64 (1.77) and 11.36 (4.02) for the young and old group respectively. Neither age, nor gender and YoE differences between the *sample 1* and the old group from the *sample 2* were found (*p* > 0.3). However YoE between old and young groups in the *sample 2* differed (*t* = 9.40, *p* < 0.001) as young subjects were recruited in an academic environment. When not specified, methods employed with both samples are assumed to be equivalent. Data from sample 2 has been partially published in previous studies of our group (Sala-Llonch et al., 2014; Vidal-Piñeiro et al., 2014).

### Neuropsychological assessment

The neuropsychological battery used comprised the major cognitive domains and included the following spanish-adapted tests (see Table 3): Mini-mental State Examination test, Rey auditory verbal learning test (RAVLT); Test de accentuación de palabras (TAP; Spanish analog of the National Adult Reading Test); WAIS-III Block design; Rey-Osterrieth complex figure (ROCF); Benton naming test (BNT); semantic and phonetic fluencies; forward and backward digits; symbol digits modalities test (SDMT), a mean d’-score of a 2 and 3-back working memory test (as in Sala-Llonch et al., 2012), Trail Making Test (TMT), Stroop test, Visual Object and Space Perception Battery (VOSP) Incomplete letters and Number locations tests and a computerized version of the Continuous Performance Test (CPT). Psychometric tests were further combined into different composite scores representing different cognitive domains (see below). Old subjects from the *sample 2* completed thorough neuropsychological batteries which are described elsewhere (Sala-Llonch et al., 2014; Vidal-Piñeiro et al., 2014).

**Table 1:** **Peak voxels of main DMN nodes. Region-of-Interests were centered in this coordinates**.

DMN node	MNI (x,y,z) sample 1	MNI (x,y,z) sample 2	Euclidian distance (mm)
**PCU/PCC**	0,−63,30	0,−63,36	6
**mPFC**	0,63,−3	0,63,−6	3
**LIPL**	−51,69,27	−48,69,30	4.24
**RIPL**	54,−60,27	51,−63,27	4.24
**LMTG**	−63,−12,−18	−66,−15,−15	5.2
**RMTG**	63,−9,−15	63,−6,−18	4.24

**Table 2:** **Sociodemographic characteristics and comorbidities factors**.

Sociodemographics and comorbidities factors			
	Distribution	Range	t/r(p)
Participants	116
Gender (male:female)	37:79	–	−1.316 (0.19)
Handedness	111:0:5	–	−0.637 (0.53)
Age range	68.25 (3.05)	63–76	−0.041 (0.67)
Years of School	11.09 (4.15)	2–21	−0.018 (0.86)
Hypertension	61:55	–	0.225 (0.82)
Diastolic pressure	76.11 (9.71)	51–103	−0.017 (0.85)
Systolic pressure	123.74 (17.34)	74–170	−0.023 (0.81)
Dyslipemia	61:55	–	−1.549 (0.12)
Total cholesterol	209.95 (35.62)	118–381	−0.150 (0.11)
Diabetes	111:5	–	−0.629 (0.629)

*In the right side of the table the relationship of these variables with mPFC-PCU DMN connectivity measures is evaluated. Gender: male:female. Handedness: Right:Left:Ambidextrous. Hypertension: Yes:No; Systolic/diastolic pressure ≥ 140/90 mmHg or antihypertensive medication. Dyslipemia: Yes:No; At least one of: (a) LDL-cholesterol ≥ 160 mg/dl, (b) HDL-cholesterol ≤ 40 mg/dl in men ≤ 50 mg/dl in women, (c) triglycerides > 150. Diabetes Yes:No; At least one of: (a) Current treatment with insulin or oral hypoglycemic drugs, (b) Fasting blood glucose > 127 mg/dl, (c) Glycated hemoglobin > 6.5%*.

**Table 3:** **Neuropsychological measures for sample 1**.

Neuropsychological measures (*n* = 116)			
Memory component		Other tests	
ROCF 3′	19.68 (6.37)	MMSE	29.22 (1.13)
ROCF 30′	19.49 (6.23)	TAP^a^	23.04 (5.83)
RAVLT total learning	48.35 (7.40)	CPT detectability	47.10 (10.49)
RAVL delayed recall	10.37 (2.49)	CPT omissions	59.60 (25.92)
**Working Memory**		Forward digits	8.80 (2.43)
d′ prime n-back (2 & 3-back)	2.12 (0.60)	Phonetic fluency (FAS)	33.97 (10.45)
Backward digits	6.40 (2.37)	TMT B^b^	104.5 (60.05)
TMTB-TMTA^b^	63.52 (45.76)	BNT^c^	54.21 (3.75)
**Speed Processing**		Semantic fluency (animals)	20.84 (5.48)
TMTA^b^	41.34 (18.30)	Incomplete letters	19.65 (0.56)
SDMT	39.62 (12.54)	Number location	9.13 (0.88)
CPT RT	60.58 (11.34)	Block design	30.92 (12.48)
**Inhibition**		FCR copy	34.33 (2.80)
CPT Commissions	46.64 (8.41)		
Stroop interference	10.84 (2.30)		

*^a^One-subject data is missing. ^b^Two-subjects data is missing. ^c^BNT scores with semantic clues*.

### MRI acquisition

All participants were examined on a 3T MRI scanner (Magnetom Trio Tim, Siemens Medical Systems, Germany) at the Center Diagnostic per la Imatge in the Hospital Clínic of Barcelona. Magnetic resonance imaging acquisition included the following sequences: a high-resolution 3D structural dataset (T1-weighted magnetization prepared rapid gradient echo [MPRAGE], sagittal plane acquisition, TR = 2300 ms, TE = 2.98 ms, 240 slices, slice thickness = 1 mm, FOV = 256 mm, matrix size = 256 × 256); a rs-fMRI sequence (T2*-weighted GE-EPI sequence, TR = 2000, TE = 26 ms, 40 slices per volume, slice thickness = 3 mm, interslice gap = 25%, FOV = 220 mm, matrix size = 128 × 128) that lasted 5 min (150 volumes); a DTI, sequence (diffusion weighted echo-planar imaging sequence; 30 directions; TR = 7700 ms; TE = 89, 60 slices, slice thickness = 2 mm, FOV = 250 mm and matrix sixe = 122 × 122) and an Pulsed-Arterial Spin labeling (PASL)-MRI perfusion acquisition (PICORE Q6T sequence, 50 tag-control scans, TR = 2500 ms, TE = 11.0 ms, T11 = 700 ms, T12 = 1800 ms, 16 slices; slice thickness = 5 mm, inteslice gap = 25%, FOV = 200 mm, matrix size = 64 × 64).

### MRI preprocessing

Magnetic resonance imaging analysis was performed using tools from FreeSurfer^1^, FSL^2^ and AFNI^3^.

*Resting-state fMRI*: Data preprocessing included removal of the first five volumes, motion correction, skull stripping, spatial smoothing (full width at half maximum [FWHM] = 7 mm), grand mean scaling, high and low pass filtering (0.1–0.1 Hz) and normalization with two-step linear transformations (Jenkinson and Smith, 2001) to a standard template. Preprocessed rs-fMRI data were further regressed with six rigid body realignment motion parameters, mean WM and mean ventricular signal. Preprocessed rs-fMRI signal was used to extract main RSNs through independent component analysis (ICA) analysis while regressed data was analyzed for calculating rs-fMRI ROI-to-ROI connectivity indices.

*Gray matter atrophy; Voxel-Based Morphometry (VBM)*: Structural data were analyzed with FSL-VBM tool (Good et al., 2001) with a standard pipeline processing (as in Balasa et al., 2012). Preprocessing included brain-extraction, tissue-type segmentation, non-linear registration to template, creation of a study-specific template and non-linearly re-registration to the study-specific template. Images were further modulated by dividing by the Jacobian of the warp field and smoothed (FWHM ≈9 mm).

*Cortical thickness*: Cortical reconstruction was performed with the semi-automatic FreeSurfer image analysis suite with high-resolution 3D images. The procedures have been described thoroughly in Fischl and Dale (2000). Reconstructed and registered individual CTh maps were smoothed using a Gaussian kernel of 15 mm FWHM and introduced into a GLM-based analysis. Two subjects were removed from further comparisons due to problems during reconstruction (*n* = 114).

*Gray-white matter contrast*: GWC was estimated with the reconstructed cortical surface by calculating the non-normalized T1-weighted image intensity contrast (100*[white–grey]/0.5*[white+grey]). Values close to 0 indicate less contrast and thus more blurring of the GM/WM boundary. GM was taken at a 0.3 projection fraction from the boundary while WM was assessed 1 mm below the WM surface. Before performing statistical analyses, the resulting GWC was mapped to a common surface and smoothed with a 15 mm FHWM Gaussian kernel. Two subjects were removed due to problems during cortical reconstruction (*n* = 114).

*White matter integrity*: Tract-Based Spatial Statistics (TBSS; Smith et al., 2006) analysis was carried out to assess WM microstructural integrity. Standard preprocessing (as in Bosch et al., 2012) included fitting to diffusion tensor model, nonlinear registration to standard space, creation of mean FA image and skeleton (thresholded at >0.2) and alignment of subjects FA images to mean FA skeleton. Nonlinear warps and the skeleton projection were applied to MD data. Four subjects were further removed due to machine artifacts (*n* = 96). The model was re-run, but this time aligning subjects to the standard skeleton instead of using the mean derived skeleton which, allowed comparisons of the results between the two samples. The results did not qualitatively change between the two procedures (data not shown).

*Perfusion imaging analysis*: Arterial Spin labeling preprocessing was carried out with FSL-BASIL toolset (Chappell et al., 2009) to obtain CBF and CBF-GM (PVE corrected) maps in absolute ml/100 g/min units. First we obtained a perfusion image by subtracting control from tag volumes and taking the average of these “difference” images. Next, kinetic model inversion (Buxton et al., 1998) was applied to the perfusion images. Constant relaxation times for blood and tissue were set at 1.6 and 1.3, inversion efficiency to 0.98 and bolus arrival time was fixed at 0.7. Spatial smoothing was applied prior to the estimated CBF images and a calculation of the equilibrium magnetization of arterial blood was then performed using WM as reference tissue. Cerebral blood flow-gray matter measures were obtained by introducing partial volume estimates in native space into the kinetic model analysis, which were calculated from native-transformed structural segmentation. The method proposed, described in Chappell et al. (2011), is able to exploit both partial volume estimates and the different kinetics of the ASL signal arising from GM and WM. Finally, CBF images were transformed to standard space.

### Data analysis

The appropriate tests were carried out with SPSS 20.0 (Statistical Package for Social Science, Chicago, IL, USA) software to handle non-neuroimaging data. Significance was set at *p* < 0.05 (Bonferroni corrected when necessary). When not specified, data is presented as mean (SD), error bars represent standard error of mean (SEM) and coordinates are reported in MNI space. In the sample 1 age, gender, and YoE were used as covariates for all analyses. Cases were excluded pairwise in the different analyses. In the sample 2 only gender was used as covariate. The rationale for not covariating for education is the overrepresentation of YoE effects due to group differences which biased the results. However, the results were not qualitatively modified when YoE was added as an additional covariate (data not shown).

#### Cognitive domain factorialization

In *sample 1*, individual psychometric tests were combined based on functional domains into four composite scales that assessed memory, speed processing, working memory and inhibition in accordance with the ageing literature (Park and Reuter-Lorenz, 2009). To compute composite scores, raw scores were converted to z-scores for all tests and then underwent factor analysis (Principal Component Analysis). Missing values (<2%) were replaced with subjects’ mean for the specified factor. The various tests that comprised each cognitive factor are shown in Table 3.

#### Definition of mPFC-PCU DMN connectivity

The measure of mPFC-PCU DMN connectivity were obtained with a two-steps pipeline that involved extraction of DMN through ICA which allow extraction of ROI coordinates for posterior ROI-to-ROI connectivity analysis. This pipeline was independently performed in both samples.

**Figure 1:**
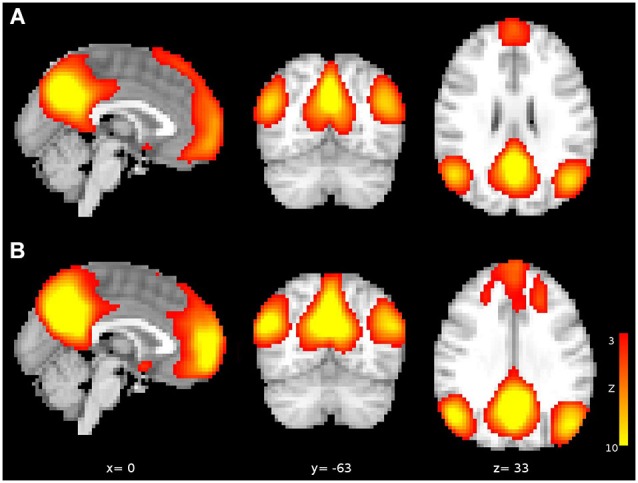
**Independent components corresponding to DMN from (A) the first sample and (B) the second sample**. Both components are arbitrarily thresholded at z = 3 and red-yellow scale indicates greater connectivity to DMN.

To obtain the DMN we performed an ICA with rs-fMRI preprocessed datasets (Beckmann et al., 2005) as implemented in the MELODIC tool which decomposes data into a number of spatial and temporal components (fixed to 20 components). From the resulting components, we identified typical components described in the literature such as right and left frontoparietal networks, a visual network, a sensoriomotor network, a cerebellum network, an executive network and the DMN, all of them common in both samples. This latter network was used for Region-of-Interest (ROI) definition. After identifying the DMN in the respective samples, which included posterior midline structures, mPFC, bilateral IPL and bilateral medial temporal gyrus (MTG) components (Figure 1), we assessed connectivity between mPFC and PCU in a ROI-to-ROI analysis that involved the definition of 6 mm-radius ROIs in peak coordinates of posterior midline and mPFC nodes. After thresholding ROIs with GM masks, the correlation between mPFC and PCU was obtained by calculating Pearson’s (*r*) correlation between preprocessed and regressed rs-fMRI ROIs time series. Next, correlations were transformed to z-scores. For sake of completeness bilateral IPL and bilateral MTG DMN ROIs were also extracted. The Euclidian distance between posterior midline structures and mPFC ROIs from both samples was small (PCU = 6 mm; mPFC = 3 mm) and mPFC-PCU correlation did not differ between old adults from the sample 2 and sample 1 population (*t* = −1.533, *p* = 0.128; see Table 1 for ROI coordinates). Correlations are reported as *r* (before z-transformations) to facilitate interpretation.

#### DMN connectivity analysis

Effects of age on DMN connectivity: Firstly, we studied whether mPFC-PCU DMN connectivity was reduced in elders. Group (young vs. old) comparisons between mPFC-PCU Z-transformed correlations were performed with the sample 2. For the sake of completeness, correlations between other DMN nodes were also performed.

Influence of DMN connectivity on cognition: To evaluate the influence of mPFC-PCU connectivity to cognition we performed a multivariate GLM-based analysis in the main sample, where connectivity, age, YoE and gender were introduced in a model predicting the four cognitive factors.

Relation of CTh, VBM, FA, MD, CBF and CBF-GM maps to DMN connectivity: To assess the relationship of structural and CBF correlates with anterior-posterior DMN connectivity, multiple voxelwise nonparametric analyses were performed. Nonparametric testing with 5000 permutations as implemented in a randomized FSL tool was used for VBM, MD, FA, CBF and CBF-GM measures followed by threshold-free cluster enhancement (TFCE) and familywise error (FWE) multiple comparison correction (*p* < 0.05). Relationships of CTh and GWC with connectivity were studied using linear modeling as implemented in FreeSurfer. After thresholding results at *p* < 0.01, FWE correction for multiple comparisons using a Monte Carlo Null-Z simulation (10.000 iterations; *p* < 0.05) was performed.

#### Correspondence of mPFC-PCU DMN correlates and patterns of age-related decline

**Figure 2:**
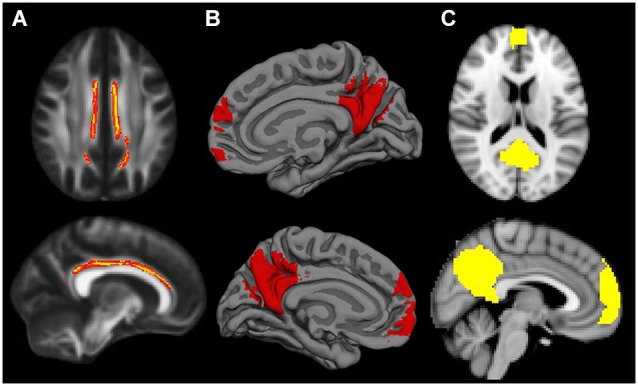
**Default Mode Network masks for the different neuroimaging modalities: (A) cingulum bundle for MD modality; (B) mPFC and posterior midline areas for GWC; (C) mPFC and posterior midline areas for VBM**. When necessary masks were resampled to match template characteristics. Cingulum mask is inflated for visual purposes.

To gain further insight into the structural correlates of mPFC-PCU DMN connectivity, we studied whether they overlapped areas of high age-related vulnerability. Consequently, these analyses were only carried out in the modalities in which significant mPFC-PCU connectivity correlations were found (MD, GWC and VBM). To do so, we extracted age-related patterns from the *sample 2* (group comparisons) and compared age-related patterns in the brain with those limited by the structural correlates of mPFC-PCU connectivity and those limited to DMN areas (and the cingulum bundle). First, we extracted maps of differences between old and young subjects using GLM-based analysis with group and gender as regressors. Further, differences in the age-related effects on whole brain, connectivity correlates and DMN areas were compared using the raw statistics obtained in the group comparison (thus age-effects) and effect size calculations were performed (Cohen’s D; Cohen, 1988). It is important to note that these tests were not inferential in nature but descriptive. Default Mode Network (mPFC-PCU) areas for the different modalities were defined as follows: for MD, the mask was defined as the conjunction of JHU white-matter tractography atlas cingulum bundle (Hua et al., 2008) and the mean sample skeleton; for VBM and GWC modalities, the DMN mask was defined as those voxels belonging to mPFC and posterior midline structure DMN areas (from the study specific DMN; *z* > 4; see Figure 2). These areas have been previously reported to be regions of high age-related vulnerability.

#### Motion correction analysis

As motion correction may affect fMRI correlations (Power et al., 2012; Van Dijk et al., 2012), mean relative translation and mean relative rotation parameters were calculated for every subject. Any subject with outlier values relative movement (>0.60 mm/degrees) was excluded from the analysis. Additionally, all significant results were re-run using these measures as covariates.

## Results

### Neuropsychological performance

Neuropsychological performance, comorbidity factors and sociodemographic characteristics of the main sample are detailed in Tables 2, 3.

### mPFC-PCU DMN connectivity is decreased in aged subjects

**Figure 3:**
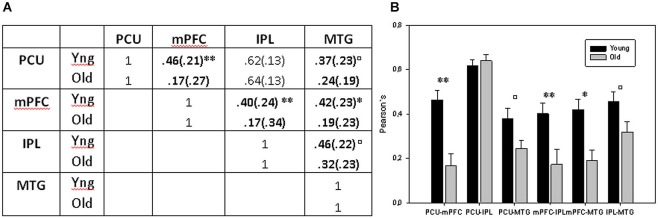
**(A)** Mean correlations between DMN ROIs. **(B)** Bar charts displaying mean connectivity between DMN ROIs in both old and young groups. ** = *p* < 0.01 and * = *p* < 0.05 after Bonferroni correction; ^□^ = *p* < 0.05 before Bonferroni correction but no longer significant when multiple comparison were applied.

First, in the between-group comparison (*sample 2*), we found reduced DMN connectivity in ageing, specifically between mPFC and PCU areas in which connectivity was clearly lower in the old group 0.17 (0.27) compared with the young group 0.46 (0.21). Inferential comparisons yielded significant differences in mPFC-PCU coupling strength (*F* = 17.858, *p* < 0.001) as a function of age. Complementarily mPFC-MTG (*F* = 12.380, *p* = 0.001) and mPFC-IPL (*F* = 7.558, *p* = 0.008) couplings also differed in both groups. PCU-MTG (*F* = 4.63, *p* = 0.037) and IPL-MTG (*F* = 4.493, *p* = 0.039) also showed significant age-related difference though they did not survive Bonferroni correction (*p* < 0.05/6 = 0.0083). No age-related differences were observed between IPL and PCU (*F* = 0.366, *p* = 0.548) ROIs (Figure 3).

### Influence of mPFC-PCU connectivity on cognition

mPFC-PCU coupling was able to predict cognitive performance as it was significantly associated with memory factor (*F* = 9.047, *p* = 0.003; Figure 5), and with speed processing domain (*F* = 3.165, *p* = 0.023), though this last comparison did not survive Bonferroni corrections (*p* < 0.05/4 = 0.0125). The working memory and the inhibitory factor were unrelated to connectivity. Years of education was associated with all cognitive factors (*F* > 13, *p* < 0.001) while age was only significantly associated with the speed processing factor (*F* = 6.482, *p* = 0.012); this was expected, given the small standard deviation in this variable.

**Figure 4:**
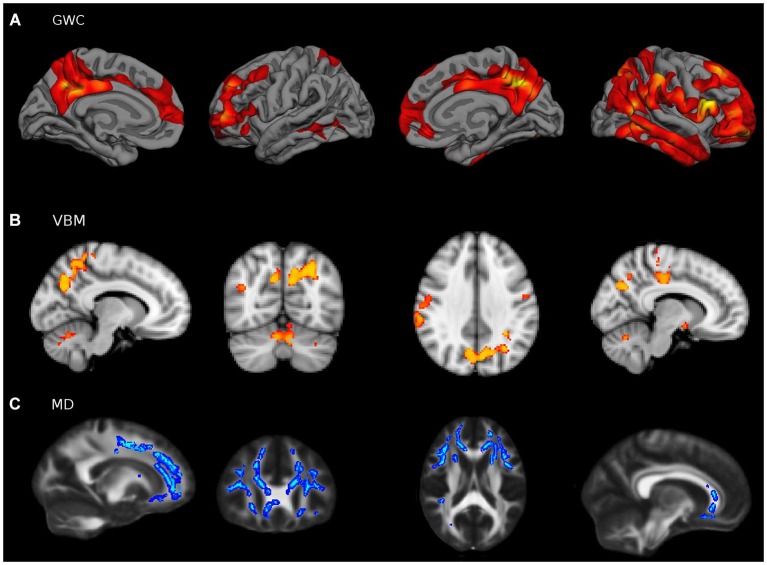
**Relationship (*p* < 0.05; FWE corrected) between mPFC-PCU DMN connectivity and (A) GWC (B) VBM and (C) MD**. Positive relationships are shown on a Red-Yellow scale and negative relationship on a blue-lightblue scale. Both VBM and CTh showed a positive correlation with DMN connectivity, thus indicating preserved GM integrity with higher connectivity values. Mean Diffusivity showed a negative relationship with mPFC-PCU connectivity thus reflecting preserved MD integrity. For visual purposes, voxel significance is depicted in clusters that survived multiple comparisons. Gray-white matter contrast image is displayed by lateral and medial views of an inflated brain. Voxel-based morphometry slices are shown at *x* = −10,10; y = −66 and *z* = 34 and Mean Diffusivity slices are obtained in *x* = −18,20; *y* = 28, *z* = 18.

**Figure 5:**
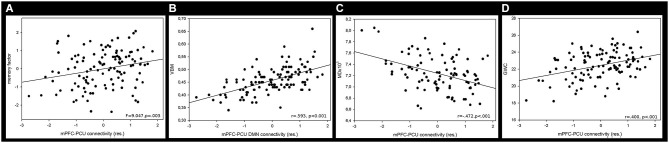
**Mean correlation between mPFC-PCU DMN connectivity and (A) memory factor, (B) VBM, (C) MD and (D) GWC**. Neuroimaging measures were obtained by calculating the mean value of the largest significant cluster (see Table 4, Figure 4).

**Table 4:** **Clusters (>100 voxels) showing relationship between GWC MD and VBM brain regions and mPFC-PCU connectivity measures**.

Modality	Size	Max	X	Y	Z	Area
GWC	35741	<0.001	48.5	−27.7	38.0	Right supramarginal
	7370	<0.001	−32.8	49.8	−0.8	Left rostral middle frontal
	5239	<0.001	−7.0	−41.5	30.6	Left isthmus cingulate
	866	0.008	−50.2	−60.7	1.1	Left inferior temporal
MD	4139	0.025	−26	29	16	Forceps minor
	3710	0.025	36	39	−2	Right inferior fronto-occipital fasciculus
	2769	0.037	19	44	21	Forceps minor
	215	0.047	−18	−3	45	Left superior longitudinal fasciculus
	158	0.045	−11	24	50	Left uncinate fasciculus
VBM	2998	0.007	−28	−38	54	Left superior parietal lobule
	1149	0.007	−38	−52	−28	Cerebellum
	302	0.017	60	−2	20	Right precentral cortex
	293	0.016	62	−32	30	Right supramarginal gyrus
	226	0.010	−50	−6	24	Left precentral gyrus
	221	0.016	14	2	−10	Pallidum
	198	0.019	68	−18	10	Right superior temporal gyrus
	125	0.035	20	−38	56	Right precentral gyrus

*See also Figure 4*.

### Relationship between structural and perfusion indices and antero-posterior DMN connectivity

*Voxel-Based Morphometry*: Regional GM intensity was positively correlated with mPFC-PCU connectivity in several areas. Most of the significant clusters were located in posterior areas of the brain. Regional GM correlations were found in the PCU, extending to the lateral occipital cortex and superior parietal lobe. Another cluster was found encompassing the right supramarginal gyrus. Other areas related to mPFC-PCU connectivity were found in temporo-occipital and temporo-parietal junctions and in the cerebellum (see Figures 4, 5, Table 4).

*Cortical Thickness*: No relationship was found between DMN connectivity and cortical thickness. However, when the threshold was less stringent (*p* < 0.05, *p* < 0.05 FWE corrected) correlations were found between mPFC-PCU connectivity and CTh in bilateral parietal and right superior frontal areas.

*Gray-White Matter contrast*: GWC correlated with mPFC-PCU coupling in a widely distributed set of regions including frontal, temporal and parietal lobes bilaterally. In the left hemisphere three clusters were found: one encompassing the middle and inferior temporal gyrus; another extending towards the PCU, isthmus and posterior cingulate; and a third cluster covered lateral prefrontal areas. In the right hemisphere, a large cluster was found covering medial and lateral parietal lobes, the lateral temporal lobe and the prefrontal cortex with peaks of significance located in pars opercularis, supramarginal and PCU regions (see Figures 4, 5, Table 4).

*Mean diffusivity*: Mean diffusivity was negatively related to mPFC-PCU connectivity thus revealing a correlation between mPFC-PCU functional connectivity and increased WM integrity. Significant clusters were found bilaterally, mainly in anterior areas of the brain and in long-range antero-posterior WM tracts. Anterior thalamic radiation, cingulate bundle, inferior fronto-occipital fascicle, uncinated fasciculus, superior longitudinal fasciculus and forceps minor were bilaterally correlated to mPFC-PCU connectivity (see Figures 4, 5, Table 4).

*Fractional Anisotropy*: In contrast to MD, no relationship was found between FA and mPFC-PCU coupling measures after multiple comparison corrections. Relaxed thresholds (*p* < 0.01) did not show relationship with mPFC-PCU DMN connectivity.

*Cerebral Blood Flow*: No relationship was found between perfusion fMRI measures and DMN connectivity. Neither uncorrected (*p* < 0.01) nor ROI analyses revealed any consistent differences.

*Gray Matter Cerebral Blood Flow*: When corrected for PVE estimates, CBF-GM estimates did not correlate with connectivity measures. No significant results were found at an uncorrected level (*p* < 0.01) or with ROI analyses.

Summarizing, both GM and WM measures were related to mPFC-PCU DMN connectivity. The topological pattern was not limited either to DMN nodes or to the main tract connecting DMN structures but was distributed across the brain in areas typically characterized as highly affected by ageing. Cerebral blood flow measures were unrelated to antero-posterior DMN connectivity while GWC, a measure of GM/WM blurring, was found to be related to connectivity in distributed brain areas. To gain further insight into these results, mPFC-PCU structural correlates were compared to the age-related patterns of decline extracted with the second sample.

### Age differences in structural modalities

**Figure 6:**
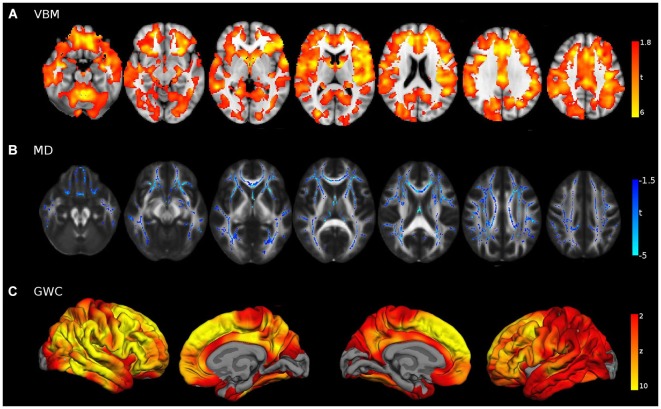
**Young vs. Old group comparisons (FWE corrected** ***p* < 0.05) in (A) VBM, (B) MD and (C) GWC neuroimaging modalities, performed with sample 2**. For visual purposes voxel significance is overlaid in those areas that survived multiple corrections. Bilateral medial and lateral surfaces are displayed in GWC while axial slices (*z* = [−20:40:10]) are displayed both for VBM and MD results.

Group comparisons with sample 2 yielded evident age-related effects in wide brain areas (Figure 6). Voxel-Based Morphometry showed a widespread pattern of decreased GM intensity in the ageing group with the exception of some regions in the occipital lobe. Areas with greatest group differences were found in the cingulate, inferior parietal and prefrontal cortices. Increased MD was also found in widespread areas of the WM skeleton in older adults, with the exception of some corticospinal tract regions which remained relatively unaffected. Maximum effects of age were identified in forceps minor, cingulum, uncinate and inferior fronto-occipital tracts. Finally, the GWC age-related pattern was strikingly significant, showing age differences in the entire cortical mantle except in primary visual areas. The greatest vulnerability to age was found in the superior and medial frontal cortices. However, lateral PFC and posterior midline structures were also highly affected, in a bilateral fashion. Overall, age-related patterns of decline showed great similitude to those reported in the literature.

### Correspondence between mPFC-PCU connectivity correlates and young vs. old differences

Structural correlates of mPFC-PCU within the primary old sample were not limited to the DMN (i.e., the underlying cortical region) or to the main tract connecting them, but extended beyond those nodes and were distributed along the cortical mantle. Interestingly, this distribution resembled the impact of age on the brain in each specific neuroimaging modality. To quantify this correspondence, mean effects of age were computed for areas related to mPFC-PCU connectivity and compared to both the overall ageing pattern and the effects of ageing in DMN areas through effect size tests.

**Figure 7:**
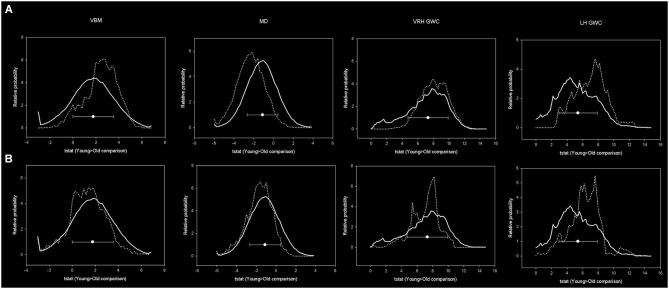
**Histograms showing distribution of age-related effects across predefined masks**. In the upper row, the continuous line represents the distribution of the age-effect across all brain voxels while dotted line represents the voxels included within mPFC-PCU correlates mask. In the lower images the dotted line represents the age-related distribution within predefined DMN areas. Bins have 0.2 width while the Y axis has been scaled to represent a relative distribution. Circles represent the means and error bars the standard deviations of age-related effects of all brain voxels.

Unequivocally, voxels that exhibited a relationship with mPFC-PCU DMN connectivity also tended to be more susceptible to the effects of age. Effect sizes were medium for VBM and right hemisphere GWC and high for MD and left hemisphere GWC according to criteria described elsewhere (Cohen, 1988). That is, for the different structural modalities, areas significantly related to mPFC-PCU DMN connectivity presented above average susceptibility to the effects of age compared to the overall pattern in their respective modality (Figure 7).

In contrast, DMN areas did not show increased susceptibility (compared to whole brain) to age either in VBM, MD or right hemisphere GWC. In left hemisphere GWC, DMN voxels presented small effects size. Finally, effect size analyses comparing age-vulnerability of DMN areas with areas related to mPFC-PCU connectivity showed, in all the modalities, an increased susceptibility to age effects in the latter regions (which showed medium effect size; Table 5). That is, structural correlates of mPFC-PCU DMN connectivity were located in areas that were more susceptible to age than DMN areas. Significantly, a proportion of voxels belonged to both areas (DMN areas and mPFC-PCU connectivity correlates, though the exclusion of these voxels did not alter effect sizes analyses). These analyses, though only descriptive, suggest that areas related to mPFC-PCU DMN connectivity are located in areas of increased vulnerability to ageing effects.

**Table 5:** **Mean effect of age and effect sizes comparisons between brain mask, mPFC-PCU correlates mask and DMN mask**.

Measure	Whole-brain age-effect	mPFC-PCU correlates age-effect	DMN age-effect	Brain vs. connectivity effect size	Brain vs. DMN effect size	Connectivity vs. DMN effect size
**VBM**	1.77 (1.89)	2.51 (1.42)	1.37 (1.65)	0.39	(−)0.21	0.73
**MD**	−1.06 (1.53)	−2.22 (1.36)	−1.42 (1.21)	(−)0.76	(−)0.24	(−)0.60
**LH-GWC**	5.38 (2.52)	7.32 (2.10)	6.49 (2.15)	0.92	0.44	0.39
**RH-GWC**	7.28 (2.52)	8.30 (1.73)	7.17 (1.68)	0.40	0.04	0.65

*Age-effects are assessed through t/z statistics and effect sizes are evaluated with Cohen’s d′. Greater age-effects represent greater differences between young and old groups*.

### Motion correction

No outliers were found. Mean relative movement was negatively associated with mPFC-PCU connectivity in both samples (*r* = −0.26, *p* = 0.006; *r* = −0.39, *p* = 0.004). However, no significant changes were found in the tests after this variable was added in the statistical models. The relationship of mPFC-PCU with memory and with age (group comparisons) remained significant (*p* < 0.05), while the relationship of mPFC-PCU connectivity with anatomical correlates was still highly significant (*p* < 0.001) in all significant clusters. While motion correction impacts connectivity, it does not significantly alter our results.

## Discussion

Main results can be summarized as follows: (1) mPFC-PCU DMN connectivity is highly reduced in ageing; (2) in ageing, this coupling is related to cognition, specifically to memory performance; (3) mPFC-PCU DMN connectivity is related to measures of GM and WM integrity in several brain areas not restricted to DMN; (4) perfusion measures are unrelated to DMN connectivity; (5) mPFC-PCU DMN structural correlates appear to be located in areas of high age vulnerability.

### DMN connectivity is reduced in ageing

Firstly, this study adds new evidence of reduced DMN connectivity in ageing assessed by rs-fMRI (Ferreira and Busatto, 2013). Reduced connectivity of mPFC node from temporal and posterior parts of DMN in aged subjects was evident even with a relatively low number of subjects (in *sample 2*). Group comparisons suggested that the most significant effect of age was present in the coupling between mPFC and posterior midline structures, in agreement with other studies (Andrews-Hanna et al., 2007; Tomasi and Volkow, 2012; Campbell et al., 2013; Mevel et al., 2013). These areas form the nucleus of DMN which is composed by other several subsystems (Laird et al., 2009) involving temporal, superior frontal and lateral parietal cortices. These results also suggest that decreases in mPFC-PCU connectivity are probably not associated with compensatory mechanisms; rather, this disconnection is likely to be a reflection of brain ageing and inefficient processing though the exact causes of age-related functional connectivity abnormalities in healthy elders are mostly unknown.

### Reduced mPFC-PCU DMN connectivity relates to worse memory function

We add further evidence highlighting the relevance of DMN resting-state connectivity over cognition in ageing. Specifically, we found a relationship between antero-posterior connectivity and a memory factor composed by visual and verbal memory scores. Significantly, in the analysis, age was used as covariate; that is, elders display a relationship between memory and mPFC-PCU coupling despite the known effect of biological age on both measures. Several other studies have investigated the effect of DMN connectivity measures and cognition in different populations, including healthy elders (Hafkemeijer et al., 2012; Ferreira and Busatto, 2013). While speed of processing (Andrews-Hanna et al., 2007) or executive (Damoiseaux et al., 2008) scores have been related to decreased DMN integrity in ageing, it is the memory domain that has been most consistently associated with DMN connectivity (Andrews-Hanna et al., 2007; Wang et al., 2010; He et al., 2012). A recent study by our group in an independent sample also found a relationship between functional DMN connectivity measures and memory using an independent sample of middle-aged and aged subjects and graph-based analysis (Sala-Llonch et al., 2014). Regional cluster coefficients were the main measure used in that study, a measure of segregation which reflects the prevalence of clustered connectivity around individual nodes. Regional cluster coefficients were significantly increased with age in numerous cortical regions, including main DMN nodes, which were negatively related to visual and verbal memory scores. This relationship persisted in posterior midline areas after regressing the effects of age, while, in addition, greater age-related connectivity changes were found between parietal and frontal areas (which include DMN and dorsal attentional network intra-connections). Despite their major methodological differences, both studies support the hypothesis that DMN rs-fMRI integrity is a key element for preserved memory encoding in ageing. In addition, the observation of decreased DMN connectivity and the pattern of decline in episodic memory on a population basis broadly coincide over the lifespan.

The specificity of DMN connectivity regarding memory performance remains to be fully elucidated though it may be related to the fact that medial temporal lobes, essential for memory processes, are often considered part of the DMN. Default Mode Network core nodes have also been strongly linked to prospective and autobiographical memory (Spreng and Grady, 2010). During rest, medial temporal lobe structures tend to be coupled with main DMN nodes; however, during episodic encoding these structures are highly activated and are disconnected respect to other DMN nodes, which are deactivated. These processes are crucial to correctly encode stimuli. Deficient coupling at rest might reflect inefficient allocation of neural resources necessary for correct performance during episodic memory tasks (Stevens et al., 2008). Surprisingly, though, most studies relating DMN with cognition highlights the role of parieto-frontal interactions (Andrews-Hanna et al., 2007; He et al., 2012; Sala-Llonch et al., 2014), and the medial temporal lobe DMN subsystem does not seem highly affected by ageing (Campbell et al., 2013), suggesting a more complex explanation linking decreases in memory and DMN connectivity. An alternative hypothesis would be to consider the antero-posterior DMN disconnection as an unspecific measure of ageing, which is altered at an early stages, probably related to the centrality of these areas regarding the connectome (de Pasquale et al., 2012), and memory as a process highly vulnerable both to age-related disruption and to cortico-cortical and cortico-limibic dysfunctions.

### mPFC-PCU connectivity is related to WM and GM integrity indices and not to cerebral blood flow

*WM microstructure*: Relationships between WM structure and DMN connectivity were evident for MD in frontal areas and in long range anterior-posterior connections which may be more sensitive to ageing effects than FA measure. In addition, GWC, thought to partially reflect WM atrophy near the GM boundary, was related to DMN connectivity.

The notion that structural networks represent the physical substrate of functional connectivity patterns in the human brain has received considerable attention over the last few years (Honey et al., 2009; van den Heuvel and Sporns, 2013). This literature suggests that the structural connectome plays a key role in the neural synchronization patterns in the human brain. However, spontaneous neural activity is not limited by the underlying anatomy, as polysynaptic connections also contribute to functional connectivity patterns (Honey et al., 2009). Several studies (Skudlarski et al., 2008; Honey et al., 2009; Horn et al., 2013) have studied brain structural-functional connectivity relationships and have demonstrated that several brain regions show similitudes between functional and structural connectivity patterns, especially those belonging to DMN areas. Regarding DMN areas, it is well established that the cingulum tract supports DMN connectivity from posterior to temporal and frontal areas (Greicius et al., 2009). Accordingly, several groups (Andrews-Hanna et al., 2007; van den Heuvel et al., 2008; Teipel et al., 2010; Khalsa et al., 2013) have reported associations between cingulum integrity and DMN functional connectivity. In particular, Andrews-Hanna et al. (2007) reported a correlation between WM integrity (including the cingulum and adjacent areas) and mPFC-PCU functional connectivity in elders. Complementarily, Teipel et al. (2010) studied the relationship between WM microstructure and posterior midline-hippocampus connectivity in aged subjects, finding that large areas of WM including the cingulum bundle were associated with DMN functional integrity. These findings have generally been discussed in terms of structural connectivity supporting functional connectivity. Nevertheless in these previous studies cingulum-DMN connectivity correlations were not especially strong after adjusting for age (*r* ≈ 0.2–0.3) and, to some extent, are comparable to those found in our study (cingulum MD—mPFC-PCU connectivity: *r* = 0.20, *p* = 0.060, data not shown). While age-related mechanisms affecting structural and functional connectivity might be partially related, the results also suggest that other mechanisms are involved in the maintenance of resting-state DMN connectivity besides those assessed with diffusion-based imaging. In development literature the link between functional and structural anteroposterior DMN connectivity seems to emerge in the late childhood (≈10 years; Supekar et al., 2010; Gordon et al., 2011). Supekar and colleagues found a DMN-like RSN in children (7–9 years) and observe an immature PCC-mPFC coupling that was unrelated to a diffusivity measure of the cingulum bundle. However, young adults displayed functional-structural connectivity relationships which lead to the suggestion that the maturation of mPFC-PCC functional connectivity depends on the maturation of WM tracts within the cingulum bundle. The emergence of DMN during development is still discussed (Power et al., 2010) but several studies suggest that late childhood is a critical period for its development. A moderating effect of age in the relationship between WM microstructure and functional communication it is probable being present in critical lifespan periods (Andrews-Hanna et al., 2007; Supekar et al., 2010) such as during development or in late adulthood where increased WM insults might critically evidence the structural-functional relationship within the DMN.

We found that DTI correlates of mPFC-PCU connectivity were not limited to the cingulum bundle, which was only related to function in its anterior portions, but extended to other WM regions associated with long-range antero-posterior tracts and frontal interhemispheric tracts. These tracts are highly affected by age (Westlye et al., 2010; Sala et al., 2012) and have been linked to cognitive processes (Bennett and Madden, 2013). In a group of young subjects Luo et al. (2012) studied structural networks associated with DMN by examining regions that covariated with those usually reported to belong to this network. Our results resemble to a great degree the structural WM networks associated with covariance networks reported in that study. Those authors suggested that long association fibers connected the GM regions that made up the DMN, suggesting a role for these fibers in forming the skeleton of the structural network underlying the DMN. Similarly, Teipel et al. (2010) found that the WM microstructure correlating with DMN functional connectivity extended to larger areas beyond the cingulum tract. As functional connectivity may be maintained in the absence of direct structural connection, a role for long-range antero-posterior fibers partially contributing to the maintenance of DMN connectivity in ageing is plausible. However none of these studies allow inferences of causality, so caution is required when discussing these relationships. Another possibility is that mPFC-PCU connectivity and WM microstructure are not strictly related by a direct structure-function relationship but may also be linked by an indirect relationship in which structural and functional connectivity are mediated by similar age-related vulnerability mechanisms.

*Gray/white matter contrast*: The rationale for including GWC contrast in our study was the strong impact of age on this measure (Magnaldi et al., 1993; Salat et al., 2009; Westlye et al., 2009) and the fact that it partially reflects WM integrity close to the brain surface. Westlye et al. (2009) suggested that, during ageing, this measure was more affected by WM values than by GM values while several authors stress that this measure reflects reduced density of myelin sheaths (Cho et al., 1997) and increased water content in WM (Magnaldi et al., 1993). It also seems that the age-related impact on WM is greater in peripheral, thinly myelinated WM (Tang et al., 1997; Bartzokis et al., 2004). This interpretation is coherent with our results, as GWC correlates of mPFC-PCU DMN largely overlapped the DMN network. Consequently, WM near GM boundaries may be closely associated with the maintenance of the synchronous function among neural networks, upon which optimal cognition would depend. These novel results are highly interesting, as might better reflect WM ageing changes, and further studies should seek to replicate them and expand them to other networks and samples. However, caution is in order, as no clear interpretation of the GWC is available as yet.

*Gray matter*: Since fMRI is generally interpreted as an indirect measure of neuronal activity, GM intensity may have a significant impact on patterns of fMRI activity (Kalpouzos et al., 2012). However, age-related decreases in DMN connectivity have been found to persist after correction for GM volume (Damoiseaux et al., 2008), thus showing that decreased resting-state activity in ageing cannot be attributed to local GM atrophy alone. Nonetheless, these results do not imply an absence of a relationship between GM and connectivity measures, as this issue has not been explicitly assessed.

Analyses of GM covariance, which allow the study of structural networks, have reported that structural GM networks mimic main RSN networks, including DMN (Supekar et al., 2010; Segall et al., 2012). Structural covariance topological patterns are thought to change during the lifespan. Specifically in the DMN, elders show a reduced pattern of GM covariance limited to posterior areas in comparison to young subjects in whom it extends to mPFC areas (Chen et al., 2011; Li et al., 2013) which is in agreement with functional connectivity studies showing deficits in antero-posterior DMN couplings. Our results showing correlations between mPFC-PCU connectivity and GM integrity limited to posterior brain areas can be explained by changes in the pattern of GM covariance with age. Posterior midline GM atrophy has usually been related to pathological ageing (Buckner et al., 2009); however, a decline in this structure is prominent in healthy aged subjects with very-low risk of dementia (Fjell et al., 2009b). Additionally, the results suggest that age-related decreases in DMN connectivity are mediated by posterior structures. This interpretation is in agreement with the assumption that the DMN primary core is located in posterior midline regions. It is plausible that the declining GM trajectories of DMN nodes become disengaged as the brain ages and the antero-posterior disconnection arises. Thus, mPFC atrophy may be unrelated to posterior midline atrophy due to both functional and structural disconnection.

*Cerebral blood flow*: In the present report, functional connectivity and CBF indices were unrelated even at uncorrected level. To our knowledge, no directly comparable studies have been published. BOLD signal changes, though, are not independent from CBF, as the ratio of oxygenated and deoxygenated hemoglobin is primarily affected by an influx of oxygenated blood in response to the increased metabolic demands of neuronal activation. Indeed, relationships with static indices of CBF and BOLD signal (Davis et al., 1998) as well as with dynamic indices have been observed (Tak et al., 2014). Evidence linking regional CBF with connectivity is limited; however, in relation to the DMN, two studies with healthy young subjects should be mentioned (Liang et al., 2013; Khalili-Mahani et al., 2014). Liang et al. (2013) linked CBF and functional connectivity strength which was stronger in DMN areas; this relationship was further discussed in terms of larger energy demands to maintain long-range connections. Khalili-Mahani et al. (2014) also found a relationship between CBF measures and DMN strength. However, that study also found that alterations in BOLD signal and rCBF differ considerably after pharmacological intervention, suggesting that these measures may provide complementary information about the dynamics of the brain’s hemodynamic responses. Studying left inferior frontal junction in ageing, Chételat et al. (2013b) did not find an association between connectivity and regional metabolism, to which CBF is closely related. Similarly, our results showed that, although both CBF and DMN connectivity are known to decline in ageing, the patterns may be unrelated.

### Topology of structural mPFC-PCU connectivity correlates corresponds to areas of high-age related vulnerability

In our study, structural correlates of mPFC-PCU DMN connectivity extended to several brain areas which were highly vulnerable to ageing effects. Importantly, mPFC-PCU connectivity correlates showed greater age-related vulnerability than DMN areas or the cingulum bundle, even though these areas have been reported to be highly vulnerable with advanced age (GM: Fjell et al., 2014a; WM: Westlye et al., 2010; Sala et al., 2012; GWC: Salat et al., 2009; Westlye et al., 2009). While these results are only descriptive and should be interpreted with caution, they highlight the value of the single measure of mPFC-PCU DMN connectivity as a metric closely related to brain ageing by linking it to structural hallmarks of lifespan brain changes.

It is possible that DMN functional connectivity alterations in elders are greater than WM microstructure and GM atrophy changes within the DMN, at least until a certain age. In this regard, some studies demonstrate clear alterations in DMN connectivity in middle-aged subjects (Bluhm et al., 2008; Biswal et al., 2010; Evers et al., 2012). In addition, although the evidence is not conclusive, it appears that GM levels in several DMN areas, especially medial temporal structures but also posterior and anterior DMN nodes (Thambisetty et al., 2010; Taki et al., 2013; Fjell et al., 2014b) as well as cingulum integrity WM indices (Westlye et al., 2010; Sala et al., 2012) may show an accelerated decline in late adulthood. While there is no doubt that brain activity is partially supported by structure integrity, it is highly feasible that, to some extent at least, functionality is affecting structure in ageing, through Hebbian principles. While plastic changes are easily assessed using rs-fMRI techniques (Albert et al., 2009), it is well known that plastic interventions, such as those produced by cognitive or behavioral demands, are able to impact both WM (Engvig et al., 2012) and GM (Engvig et al., 2010) structure assessed with MRI techniques. The suggestion that regions characterized by a high degree of life-long plasticity are vulnerable to the detrimental effects of normal ageing (and that this age-vulnerability also renders them more susceptible to additional, pathological AD-related changes, Mesulam, 1999; Fjell et al., 2014a) is a provocative hypothesis that is gaining attention and is coherent by our results. The relevance of plasticity has been both considered as an adaptive mechanism and as a factor of risk that may lead to pathology (Oberman and Pascual-Leone, 2013). Default Mode Network areas are among the ones with the highest neuroplasticity in the cerebral cortex, playing a central role in brain functioning as well as being highly involved in learning and memory processes, which implies greater demands of plasticity. This increased demand of activity and/or life-long plasticity mechanisms may thus make this system especially vulnerable during the lifespan (Fjell et al., 2014a). Within this context, and through its association with GM and WM areas showing high age-related changes, dysfunctional DMN connectivity may represent an early marker of brain ageing, which could emerge from maladaptive plasticity mechanisms during the lifespan.

### Limitations

The study has a number of limitations. First, the results may be partially explained by factors that were not assessed such as a proportion of cases fulfilling preclinical AD criteria by virtue of an altered early biomarker (i.e., high levels of amyloid-β (Aβ) deposition), the presence of the APOE ɛ4 allele or dopamine depletion. Around 20% of older subjects without cognitive decline have significant Aβ deposition, an early AD biomarker (Chételat et al., 2013a). Amyloid-β has been hypothesized as a potential cause of rs-fMRI abnormalities (Ferreira and Busatto, 2013), such as reductions in DMN connectivity (Hedden et al., 2009), further diminished in AD (Jones et al., 2011), in a normalcy-pathology homology. However, decreases in functional connectivity exist without evidence of Aβ deposition (Andrews-Hanna et al., 2007) as well as it is evident in middle-aged subjects which have a small percentage of Aβ+ subjects. Also, most mPFC-PCU connectivity correlates results were unrelated to areas proposed as AD biomarkers. While it is unlikely that the results can be explained by Aβ deposition, partial contamination of the results cannot be ruled out. Further studies might assess a possible moderator or mediator effect of Aβ deposition in the reported relationships or alternately might use samples of subjects with low probability of dementia. Similarly, dopamine depletion and genetic polymorphisms such as APOE ɛ4 may influence antero-posterior DMN connectivity (Achard and Bullmore, 2007; Sheline et al., 2010) and further interact with multimodal and cognitive correlations. However, the effect of the dopamine system on the DMN seems weak (Achard and Bullmore, 2007) in contrast to fronto-striatal circuits, while the exact effect and direction of the APOE ɛ4 allele on DMN connectivity is still debated (Reinvang et al., 2013).

Another potential limitation is the effect of motion over rs-fMRI measures, however, *post hoc* tests did not qualitatively change any of the main results reported here. *Post hoc* testing was used because no standardized procedures exist to deal with this potential confounding. A third limitation refers to the analyses performed to assess age-related decline, since relatively small group comparisons were carried out. Using this methodology instead of cohorts encompassing the entire life-span or longitudinal approaches may have altered age-related patterns. Still, the patterns were topologically similar to those reported previously in the literature and we preferred to exploit the advantages of using a sample acquired in the same scanner machine. Lastly, the exploratory and correlational character of the study limits the explanatory power of the results.

## Conclusions

This study provides valuable new information of DMN connectivity in healthy ageing. The main finding is that mPFC-PCU DMN connectivity is related to GM and WM indices of integrity, not only in DMN areas but also in areas of high age-related vulnerability. This coupling is affected by ageing and is related to cognitive performance in elders. Elucidating the basis of disconnection in ageing is of the utmost importance for understanding healthy brain function and cognition as well as the development of brain pathology.

## Conflict of interest statement

The authors declare that the research was conducted in the absence of any commercial or financial relationships that could be construed as a potential conflict of interest.

## Footnotes

^1^http://surfer.nmr.mgh.harvard.edu, version 5.3.

^2^http://www.fmrib.ox.ac.uk/fsl/, version 5.0.6.

^3^http://afni.nimh.nih.gov/
